# Diverse polyketides from the marine endophytic *Alternaria* sp*.* LV52: Structure determination and cytotoxic activities

**DOI:** 10.1016/j.btre.2021.e00628

**Published:** 2021-05-24

**Authors:** Manar M. Mahmoud, Ahmed S. Abdel-Razek, Hesham S.M. Soliman, Larissa V. Ponomareva, Jon S. Thorson, Khaled A. Shaaban, Mohamed Shaaban

**Affiliations:** a Pharmacognosy Department, Faculty of Pharmacy, Helwan University, Helwan City-Cairo 11884, Egypt; b Organic and Bioorganic Chemistry, Faculty of Chemistry, Bielefeld University, d-33501 Bielefeld, Germany; c Microbial Chemistry Department, Division of Genetic Engineering and Biotechnology Research, National Research Centre, El-Buhouth St. 33, Dokki-Cairo 12622, Egypt; d Center for Pharmaceutical Research and Innovation, College of Pharmacy, University of Kentucky, Lexington, KY 40536, United States; e Department of Pharmaceutical Sciences, College of Pharmacy, University of Kentucky, Lexington, Kentucky 40536, United States; f Chemistry of Natural Compounds Department, Division of Pharmaceutical Industries, National Research Centre, El-Buhouth St. 33, Dokki-Cairo 12622, Egypt; g Pharm D program, Egypt-Japan University of Science and Technology (E-JUST), New Borg El-Arab City, 21934 Alexandria, Egypt

**Keywords:** Polyketides, Marine endophyte, Alternaria sp., Taxonomy, Cytotoxicity

## Abstract

We report the isolation and characterization of five polyketides [alternariol (**1**), alternariol-9-methyl ether (**2**), altertoxin I (**3**), altertoxin II (**4**) and tenuazonic acid (**5**)] from the marine endophytic *Alternaria* sp. LV52 derived from *Cystoseira tamariscifolia,* collected from the Red Sea at Nabq-Bay, Egypt. The chemical structures of compounds **1–5** were identified by extensive 1D, 2D NMR, and HR mass measurements. Isolation and phenotypic and genotypic characterization of the producing fungus is reported. The antimicrobial activity of the produced extract and derived compounds was examined against a panel of test organisms. In addition, an *in vitro* cytotoxic activity of **1–5** was performed against diverse cancer cell lines: HEPG2, HELA, A549 and PC3, revealing that compounds **2** and **4** are potentially cytotoxic against A549 and PC3 with EC_50_ of 0.73 µg/ml (2.69 µM) and 0.17 µg/ml (0.64 µM) for **2**, and 0.40 µg/ml (1.15 µM) and 0.12 µg/ml (0.33 µM) for **4**, respectively.

## Highlights

• Isolation and characterization of five polyketides **1–5** from the marine endophytic *Alternaria sp.* LV52 derived from *Cystoseira tamariscifolia*.
• The chemical structures of compounds **1–5** were identified by extensive 1D, 2D NMR, and HR mass measurements, and by comparison with literature data.
• Isolation and taxonomic characterization of the producing fungus is intensively reported on the bases of morphological and genotypic analysis.
• The antimicrobial of the produced extract and derived compounds were examined against a panel of test organisms.
• Compounds **2** and **4** are potentially cytotoxic against human non-small cell lung (A549) and prostate (PC3) cancer cell lines.

## 1 Introduction

Fungi offer enormous biodiversity, with around 70,000 known species, and an estimated 1.5 million species in total [1,2]. Accordingly, fungal strains represent a rich source of natural products with wide-ranging biological activity [3]. Natural products from fungal sources have been employed as pesticides, herbicides, antibiotics, immunosuppressants, anti-infectives, and anticancer agents [[4], [5], [6], [7], [8], [9], [10]]. Nearly 80% of the world's antibiotics have been originated from microbial sources obtained from soils around the world [6]. However, new microbial habitats, particularly, the ‘marine ecosystem’, which supports an array of novel microbial diversity [11,12], are required to be studied for microbiota that produce useful bioactive compounds able to overcome the newly explored infectional diseases. In particular, habitats such as those in which the environment is extreme in hotness [13], pressure or salinity, and the endophytes are representing further promising sources of microorganisms [12,[14], [15], [16]]. The existence of endophytes has been known for over a hundred years, but a study on their bioactive products is relatively sparse, until recently [12,17]. The role of endophytes in drug discovery began in the 90 s with the isolation of taxol, the world's first billion-dollar anticancer drug, and led to an explosion in endophytic studies [18,19]. Consequently, a diverse array of novel, eco-friendly secondary metabolites comprising bioactivities (anticancer, antioxidant, antifungal, antibacterial, antiviral, insecticidal, and immunosuppressant) have been procured from endophytes [18,[20], [21], [22]].

**Fig. 1:**
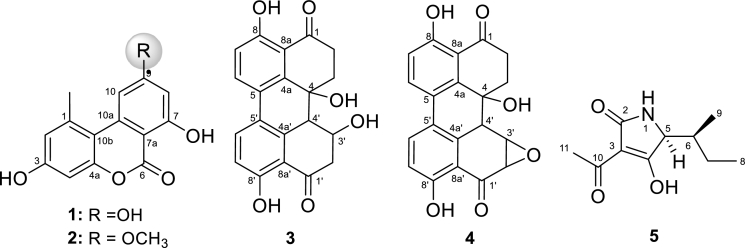
Chemical structures of compounds **1–5**, produced by the endophytic *Alternaria* sp. LV52.

In the course of our searching for bioactive polyketides possessing anticancer activities from endophytes, a chemical investigation of the marine endophytic *Alternaria* sp. cultivated on rice solid media delivered alternariol (**1**), alternariol-9-methyl ether (**2**), altertoxin I (**3**), altertoxin II (**4**) and tenuazonic acid (**5**) (Fig. 1). The chemical structures of compounds **1–5** were determined by extensive 1D and 2D NMR spectroscopy, HR-ESI mass measurements, and by comparison with literature data. Biologically, the antimicrobial activity of the crude extract and isolated compounds was studied using a panel of test microorganisms. Furthermore, the cytotoxic activity of the fungal extract compared with the obtained bioactive metabolites was evaluated against several cancer cell lines, namely KB-3–1, HEPG2, HELA, A549 and PC3**.**

## 2 Experimental section

### 2.1 General experimental procedure

NMR spectra (^1^H NMR, ^13^C NMR, DEPT, COZY, HMQC, and HMBC) were measured on Bruker Avance DRX 500 spectrometer using standard pulse sequences and referenced to residual solvent signals. ESI-HRMS was determined using GCT Premier Spectrometer. Column chromatography was carried out on silica gel 60 (0.040–0.063 mm, Merck) and Sephadex LH-20 as the stationary phases. Preparative TLC (0.5 mm thick) and analytical TLC were performed with pre-coated Merck silica gel 60 PF_254+366_. *R*_f_ values and visualization of chromatograms were carried out under UV light (*λ = *254 and 366 nm) and further by spraying with anisaldehyde/sulphuric acid followed by heating.

### 2.2 Isolation of the producing strain

The strain *Alternaria* sp. LV52 was obtained from the marine algae *Cystoseira tamariscifolia*. The hosting algae sample was aseptically rinsed twice with sterile seawater followed by sterilization with 70% EtOH for 30 s then washed with sterile seawater. Subsequently, the marine organism was aseptically cut with a sterile scalpel to reach the inner tissue surface then transferred to a conical flask (100 ml) containing 50 ml sterile seawater and agitated using a reciprocal water bath (30 °C) for 30 min. The prepared suspension was subjected to serial dilutions (10^−1^ to 10^−6^) and aliquots (0.1 mL) of serially diluted samples were used to inoculate Petri dishes containing PDA medium (Agar 15 g\L, dextrose 20 g\L, potato extract, 4 g/L). The plates were then incubated for 6 weeks at 30 °C. The colonies with distinct morphological characteristics were selected and transferred onto freshly prepared solid media (slants) and stored in a refrigerator at 4 °C until use [23]. The colonies with distinct morphological characteristics were selected and transferred onto freshly prepared solid media and stored in a refrigerator at 4 °C until use. The strain is deposited at Microbial Chemistry Department, National Research center (NRC), Egypt.

### 2.3 DNA isolation and 18S rDNA sequencing

For the analysis of fungal genomic DNA, bead beating-extracted DNA and spin-column purification by ABT DNA mini extraction kit (Applied Biotechnology Co. Ltd., Ismailia, Egypt) have been carried out. Briefly, the fungal spores were suspended in 200 µL of distilled water containing zirconia silica beads, then homogenized by vortex mixing. Subsequently, the mixture was incubated at 100 °C for 15 min. Furthermore, the lysis buffer and proteinase K were added to the sample under investigation, and DNA extraction was preceded according to manufacturer instructions. The genomic DNA was investigated using fungi-specific primer set ITS1 (5′-TCCGTAGGTGAACCTGCGG- 3′)/ITS4 (5′-TCCTCCGCTTATTGATATGC-3′) to amplify 18S rRNA gene and ITS (Internal Transcript Spacer) regions. The following amplification profile was used: an initial denaturation step at 95 °C for 5 min was followed by 35 amplification cycles of 55 °C for 30 s, 72 °C for 90 s, and a final extension step at 72 °C for 3 min. The PCR products were detected and visualized by (UV) fluorescence after ethidium bromide stain. The produced sequences of the fungal strain were aligned and recorded on GenBank database. The aligned sequence was used to construct the phylogenetic tree using MEGA X software.

### 2.4 Morphological and cultural characterization of strain LV52

The macro- and micromorphology of the strain LV52 were studied on a culture grown at 37 °C for 10 days on PDA medium. The cells were examined under a light microscope (Olympus CH-2) at a magnification of (1200 ×) at National Research center (NRC), Egypt.

### 2.5 Fermentation, working up, and isolation

The spore suspension of the fungal strain was inoculated into 100 mL of ISP2 medium composition: malt extract, 10 g l^−1^; yeast extract, 4 g l^−1^, and glucose, 4 g l^−1^ at 30 °C for 3 days as seed culture. 5 mL of seed culture were used to inoculate 1 L Erlenmeyer flasks (6 flasks) containing rice medium composition: 100 g commercial rice; 100 mL of 50% seawater containing 5% peptone. The flasks were incubated for 14 days at 37 °C. After harvesting, the fungus cultures were soaked in methanol (200 ml for each flask) followed by vigorous shaking for 1 hr. The obtained methanol extract was then separated from rice by filtration under vacuum. After filtration, the water/methanol fraction was evaporated to remove methanol using a rotary evaporator (Heidolph) [24]. After complete evaporation of methanol, the water phase was re-extracted by ethyl acetate. The obtained ethyl acetate extract was finally *in vacuo* concentrated to dryness. An application of crude extract of *Alternaria alternate* (6.0 g) to column chromatography using silica gel column (2 cm × 60 cm) eluted with cyclohexane-DCM-methanol and monitoring by TLC has afforded five fractions: F1 (1.0 g), F2 (1.0 g), F3 (0.6 g), F4 (1.5 g), F5 (0.5 g). The non-polar fractions (F1-F2) were ignored as they were of fats nature. Fraction 3 was subjected to Sephadex LH-20 (MeOH) column yielding a major crude product (210 mg) of alternariol-9-methyl ether (**2**) which has been further purified on a second Sephadex LH-20 column (MeOH) yielding 130 mg as colorless solid of the latter (HPLC-Purity: 98%). Purification of fraction 4 on silica gel column (1.5 cm × 30 cm) eluted with DCM-MeOH gradient resulted in three sub-fractions 4A (0.5 g), 4B (0.4 g) and 4C (0.4 g). Sub fraction (4A) afforded a major crude yellow oil (400 mg) of tenuazonic acid (**5)**, which was further purified on Sephadex LH-20 (MeOH) to be afforded as faint yellow of 350 mg yield (HPLC-Purity: 98%). Sub-fraction (4B) was re-purified on Sephadex LH-20 (MeOH) as well to afford altertoxine II (**4**, 12.0 mg, HPLC-Purity: 99%) as a brownish-orange solid. The third sub-fraction (4C) was likely purified, resulting in a colorless solid of alternariol (**1**, 35 mg, HPLC-Purity: 97%). Fraction 5 was finally subjected to purification by Sephadex LH-20 (DCM:MeOH, 1:1) followed by PTLC (DCM/5%MeOH) to give orange crystals of altertoxine I (**3**, 22 mg, HPLC-Purity: 99%). Details of physico-chemical properties and spectral data of compounds **1**–**5** are shown in the supplementary file.

### 2.6 Biological activity

#### 2.6.1 Antimicrobial activity assay

Antimicrobial activity testing of the fungal crude extract and the isolated compounds were carried out against a set of microorganisms using paper-disk diffusion assay [25] with some modifications according to our previous work [26].

#### 2.6.2 Cytotoxicity assays

Methodology of the cytotoxic assaying of the fungal extract and obtained compounds against the human cervix carcinoma cells KB-3–1 was carried out according to our previous work [26,27]. Cytotoxic assaying against Liver (HEPG2) and cervical (HELA) cancer cell lines was carried out according to [28] methodology. Cytotoxicity (human non-small cell lung cancer cell A549 and prostate cancer cell PC3) assays were accomplished in triplicate following previously reported protocols [[29], [30], [31]]. Vehicle (DMSO) was used as the negative control and actinomycin D (A549 and PC3) was used as a positive control.

## 3 Results and discussion

### 3.1 Taxonomic characterization of the producing strain

**Fig. 2:**
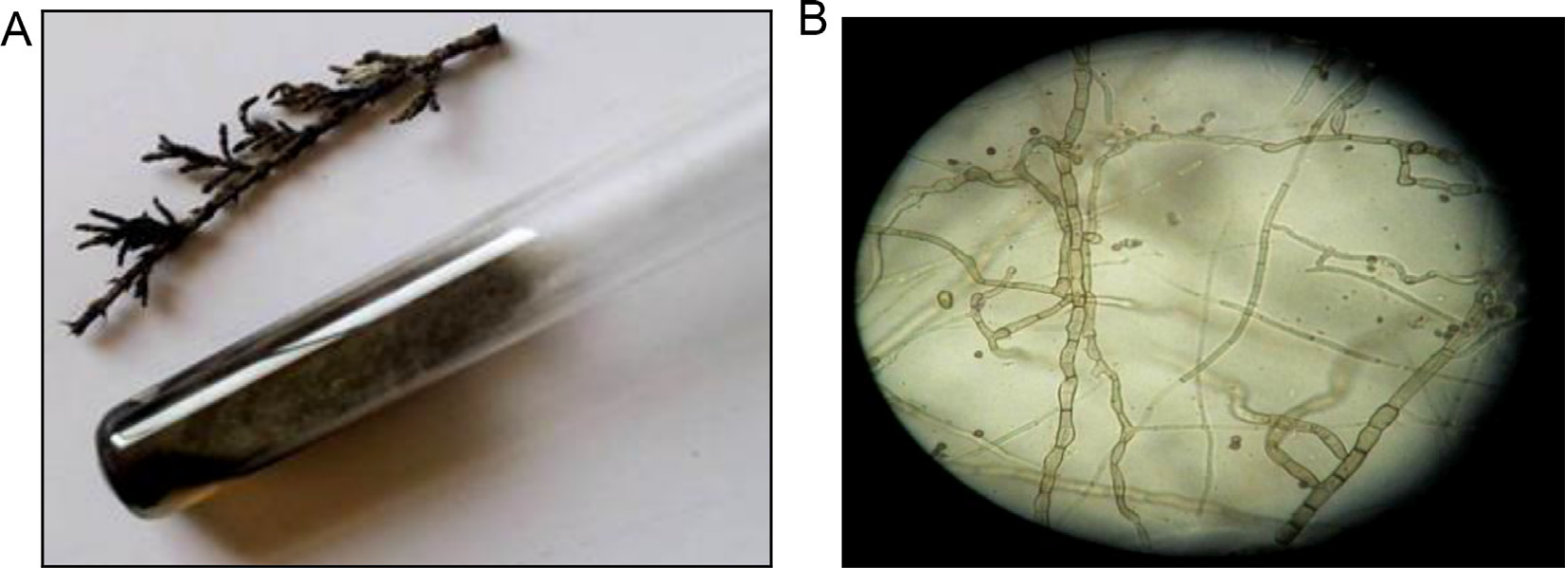
A. Photo showing 10-days age of LV52 isolate cultivated slant on PDA medium and the morphology of *Cystoseira tamariscifolia*, Figure 2B Light microscopy of *Alternaria alternate*.

**Morphological Characterization:** Colonies are fast-growing in grayish-black color. Conidia appear under light microscope as short, brown multicellular and produced from simple, branched, short elongate conidiophores (Figs. 2A-B).

**Fig. 3:**
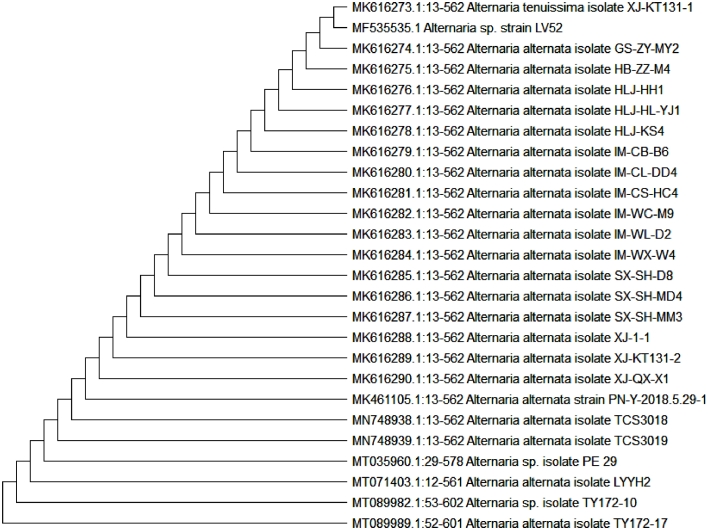
Neighbor-joining phylogenetic tree of strain Alternaria sp. LV52 based on its 18S rRNA gene sequence, showing its close relationship to *Alternaria* spp.

**Genotypic identification**: The purified PCR product of LV52 isolated genomic DNA using a specific primer has been sequenced. The produced 18 s rRNA sequences were aligned using BLAST and the phylogenetic tree was constructed using MEGA X software revealing that the isolate has a close similarity to Alternaria spp. The sequence has been recorded on the GenBank database (accession no. 535,535) and the isolate has been assigned as *Alternaria* sp. LV52 (Fig. 3).

### 3.2 Fermentation and structure elucidation

The fungus was cultured on a solid rice medium. The generated fungal extract exhibited numerous bands on TLC with a wide polarity range. Separation of the obtained fungus extract using a series of chromatographic techniques afforded five diverse polyketides: alternariol (**1**), alternariol-9-methyl ether (**2**), altertoxin I (**3**), altertoxin II (**4**) and tenuazonic acid (**5**). The chemical structures of the obtained compounds were identified by intensive studies of NMR (1D and 2 D) and HR-MS as described below.

According to the productivity profile, the obtained metabolites from the fungus extract (6.0 g) have been arranged as tenuazonic acid (350 mg; 5.83%) > alternariol-9-methyl ether (130 mg; 2.16%) > alternariol (35 mg; 0.58%) > altertoxin I (22 mg; 0.36%) > altertoxin II (12 mg; 0.2%) from the productivity point of view, with a purity in the range of 95–99% according to HPLC-analytical control.

Alternariol (**1**) and alternariol-9-methyl ether (**2**) were obtained as colorless solids with UV absorbance (254 nm) and blue fluorescence (366 nm) which turned pink on spraying with anisaldehyde/sulphuric acid. The molecular formula and molecular weights of **1** and **2** were established as C_14_H_10_O_5_ (258) and C_15_H_12_O_5_ (272), respectively, by HRESI (with a corresponding 10 DBE). Based on the analyses of ^1^H NMR/H,H COZY spectra, compounds **1** and **2** showed four *meta*-coupled protons, being for two individual aromatic residues, an aromatic bounded methyl, along with a methyl ether in **2** instead of a free phenolic group in **1**. According to ^13^C and HSQC NMR fourteen and fifteen carbon signals were assigned for **1** and **2**, respectively. The full NMR assignments of both compounds (**1, 2**) were finally confirmed using HMBC connectivities (see supplementary data, Figure S1, **Error! Reference source not found.** S1), deducing their structures as alternariol (**1**) and alternariol-9-methyl ether (**2**), respectively [32]. Biologically, alternariol derivatives have been reported with strong antimicrobial, remarkable antinematodal [33], and potent antiviral activities [34].

Altertoxin I (**3**) and altertoxin II (**4**), two additional polyketides of different nature than those of **1** and **2,** were obtained as yellow solids exhibiting greenish-yellow fluorescence bands (at 366 nm) and turned to brown pigmentation on exposure to anisaldehyde/sulphuric acid and heating. The molecular weights of **3** and **4** were confirmed by ESI MS as 352 and 350 Daltons, with corresponding molecular formulas C_20_H_16_O_6_ and C_20_H_14_O_6_ of DBE 13 and 14, respectively.

According to ^1^HNMR/^1^H-^1^H COZY spectra, both compounds displayed high similarity in their resonating structures. They observed two *peri‑*hydroxy groups (12.64/12.27 in **3** and 12.66/11.95 in **4**) in respect with benzenoid carbonyls, four doublets of *o*-coupled aromatic protons, an ethanediyl group (3.11/2.64, 2.95/2.38 in **3**, and 3.12/2.65, 2.84/2.47 in **4**), in addition to a non-oxygenated methine at 3.02 and 3.50, respectively. The sole difference between **3** and **4** structures was attributed to the creation of oxirane ring between the oxymethine at 4.70 and the neighbor methylene 2.86/3.05 in **3** forming an epoxy group of two oxy-methines at 4.31 and 3.60 in **4**. Based on ^13^C and HSQC spectra (see supplementary data, Table S2), both compounds have the same number of carbon signals, meanwhile, the three-membered ring (epoxide) in **4** was formed at where the oxygenated methine (66.1) and neighbor methylene carbon (47.8) in **3** were upfield and downfield shifted to 56.1 and 52.7, respectively in the epoxide ring of **4.** The full assignment of both compounds (**3** and **4**) was finally deduced using HMBC experiment (see supplementary data, Figure S1), confirming their structures as altertoxin I (**3**) and altertoxin II (**4**), respectively [35]. When these compounds were tested at concentrations insignificantly toxic to T-cells, altertoxins I (**3**) and II (**4**) were reported as potential inhibitors of replication of the HIV-1 virus [36]. Altertoxin II (**4**) has been reported as a potent mutagen and DNA strand-breaking agent [37].

Tenuazonic acid (**5**), as the final polyketide produced by the studied fungus, was obtained as the major metabolite in the organic extract with UV absorbance (254 nm), which turned to brown staining on spraying with anisaldehyde/sulphuric acid. Its molecular formula was deduced as C_10_H_15_NO_3_ with a molecular weight of 197 Dalton and 4 DBE. According to ^1^H and H,H—COZY spectra, structure **5** displayed an acidic broad singlet at 13.07 along with three methyl signals of singlet (2.39), doublet (1.91), and triplet (0.83) pattern, concluding the presence of an aromatic/acetyl bounded methyl and an isopentyl substructure, at where the latter displayed multiplet methylene (1.17, 1.31; CH_2_–7) and two methine protons at 1.91 and 3.75 (see supplementary data, Table S3). Based on ^13^C NMR/HSQC spectra, 10 carbon signals were observed, being for three methyl, two methine, one methylene groups in addition to four *sp*^2^ fully substituted carbons, among them three carbonyls (195.8, 184.2, and 175.7) (see supplementary data, Table S3). A final assignment of the structure using HMBC experiment (see supplementary data, Figure S1, Table S3) confirming it as tenuazonic acid (**5**) [38].

During an antiviral screening program, culture filtrates of an *Aspergillus,* tenuazonic acid was found to inhibit the cytopathic effect of measles virus in HEp2 cell cultures and to exhibit antibacterial activity referable to penicillins [39]. In a relatively recent study, isolation of dichloromethane extract of *Alternaria alternata* identified tenuazonic acid as potentially active against *Mycobacterium tuberculosis* H37Rv [40].

### 3.3 Biological activity

Based on paper disk antimicrobial assaying in comparison with gentamycin, the fungal extract possessed a low to moderate activity against diverse microbial pathogens: *Pseudomonas aeruginosa, Staphylococcus aureus, Bacillus subtilis, Candida albicans,* and *Saccharomyces cerevisiae* (Table 1). Interestingly, tenuazonic acid (**5**) was the sole active compound against all test organisms among the isolated compounds (**1–5**), meanwhile compounds **1**–**4** were inactive against the tested microorganisms up to 1 mg/disk (25 µL disk^−1^) concentration.

**Table 1:** Antibacterial and antifungal activity of crude extract produced by *Alternaria* sp. LV52 and compound **5** (conc. 40 mg/mL, 25 *µ*Ldisk^−1^ (≡1 mg disk^−1^ [*ϕ* 5 mm]).

Test organism	Crude Extract	Tenuazonic acid (**5**)	Gentamycin
*Bacillus subtilis*	8	9	16
*Candida albicans*	8	8	15
*Pseudomonas aeruginosa*	9	11	18
*Saccharomyces cerevisiae*	7	8	18
*Staphylococcus aureus*	–	9	20

According to literature assessment, alternariol derivatives showed moderate to weak antimicrobial activities against *S. aureus, B. subtilis, P. aeruginosa*, and *C. albicans*. In particular, alternariol is the most active against *B. subtilis* with MIC value of 8.6 µg/mL, meanwhile alternariol 9-methyl ether is not sensitive at the concentration of 50.0 µg/mL, indicating methylation of 9-OH may be detrimental for the antibacterial activity [41]. Altertoxin I showed weak antimicrobial activity against tested strains [42]. Finally, tenuazonic acid exhibited a moderate antibacterial activity against Gram-positive bacteria.

Alternaria toxins have been reported to show high cytotoxic activity against mammalian cells as well as teratogenicity in mice fetuses [43]. Some individual mycotoxins such as alternariol (AOH) (**1**), alternariol methyl ether (AME) (**2**), though not acutely toxic, are mutagenic and genotoxic in various *in vitro* systems. Tenuazonic acid (**5**) has been deemed to be the highest toxic among the Alternaria toxins and has been proven to be toxic to several animal species [43].

**Fig. 4:**
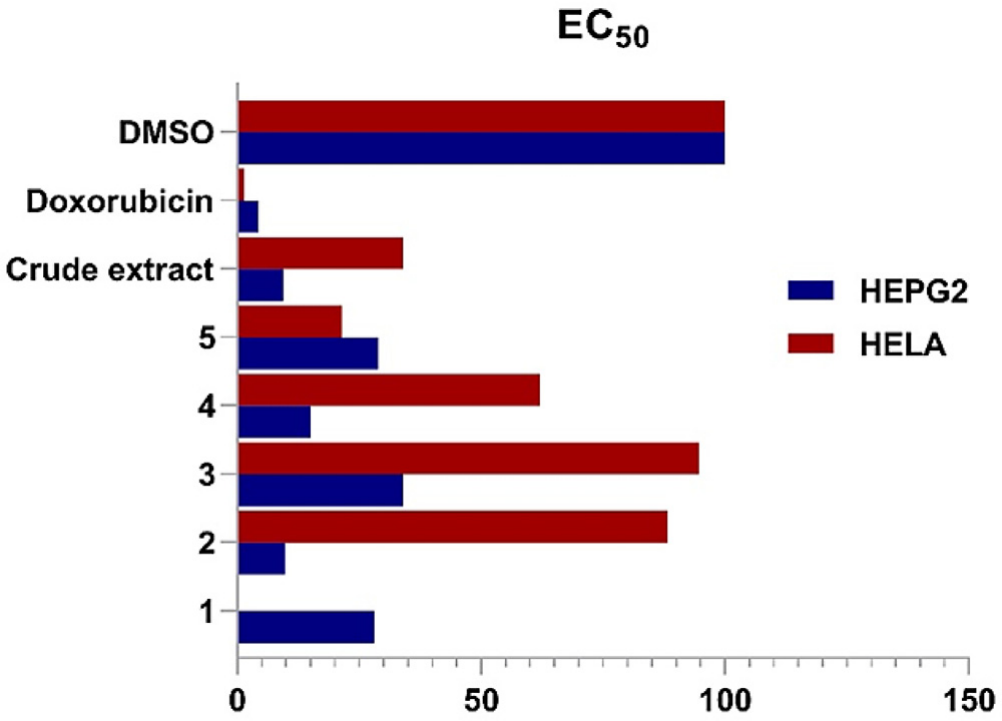
Chart showing the summary of EC_50_ for compounds **1**–**5**, crude extract and control (Doxorubicin) against **HEPG2:** EC_50_ for compounds [**1 (**28 µg/ml), **2 (**9.8 µg/ml), **3 (**34 µg/ml), **4 (**15 µg/ml), **5 (**28 µg/ml), **crude extract** (9.34 µg/ml) and **Doxorubicin** (4.28 µg/ml)]. **HELA:** EC_50_ for compounds **[2–4** (>50 µg/ml)**, 5 (**21.5 µg/ml), **Crude extract** (34 µg/ml) and **Doxorubicin** (1.45 µg/ml)]. Compound **1** was not tested against HELA cell line due to the lack of material. For more details, see Supporting Information, Table S4 and Fig. S32.

**Fig. 5:**
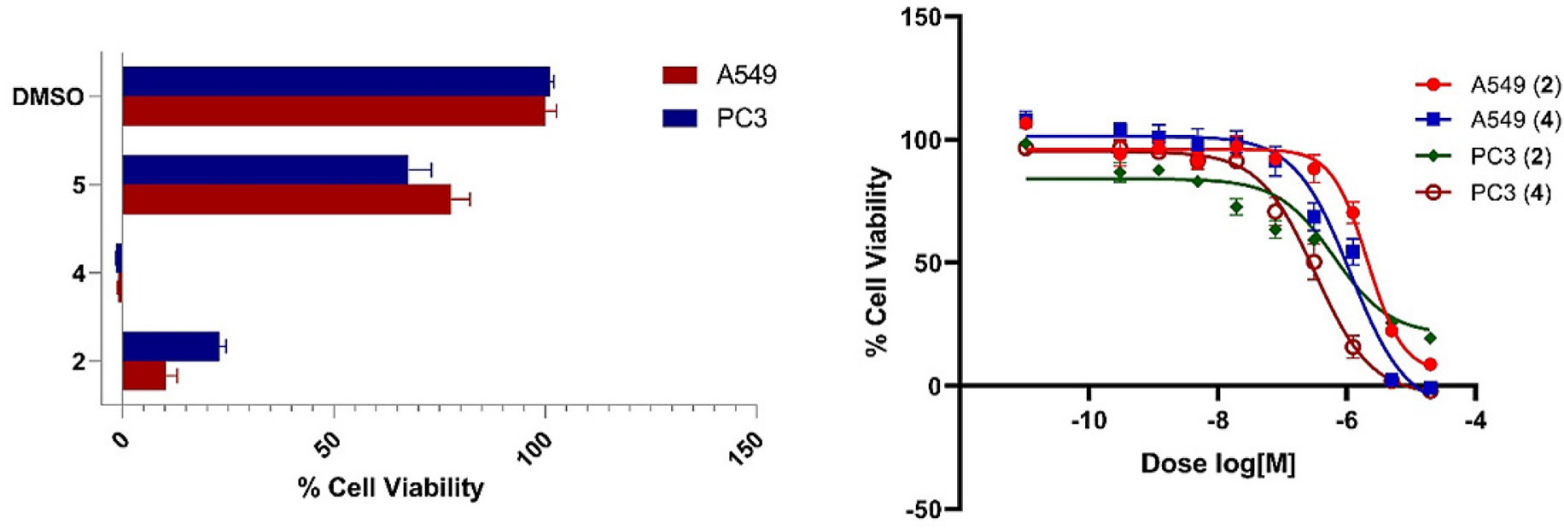
**A)** Cytotoxic activity shown as a% Cell Viability of A549 (non-small cell lung cancer) and PC3 (prostate cancer) human cell lines at 80 µM of compounds **2** (21.76 µg/ml), **4** (28 µg/ml) and **5** (15.76 µg/ml). **B)** Dose-response of compounds **2** and **4** against A549 (non-small cell lung) and PC3 (prostate) human cancer cell lines (72 h treatment). **A549:** EC_50_ for compounds **2** [0.73 µg/ml (2.69 µM)] and **4** [0.40 µg/ml (1.15 µM)]. **PC3:** EC_50_ for compounds **2** [0.17 µg/ml (0.64 µM)] and **4** [0.12 µg/ml (0.33 µM)].

In the current study, the *in vitro* cytotoxic activity of LV52 crude extract was carried out against KB-3–1 cells exhibiting cytotoxic activity of IC_50_ 0.11 mg/mL, using griseofulvin (19 µmol/l) as a positive control. The *in vitro* cytotoxic activity of the strain extract and corresponding compounds was carried out against HepG2 and HELA cell lines (Fig. 4 and Supporting Information, Table S4 and Figures S32A-B). In accordance, the extract showed an EC_50_ of 9.34 µg/mL and 34 µg/mL, respectively. Alternatively, compounds **1–5** showed potent cytotoxicity against HepG2 of EC_50_ 9.8, 28, 34, 15, and 28.8 µg/mL, respectively (Fig. 4 and Supporting Information, Table S4, and Figure S32A). In contrast, tenuazonic acid (**5**) was the sole one among the isolated compounds, which showed cytotoxicity against HELA cell line (EC_50_ 21.5 µg/mL) (Fig. 5 and Supporting Information Table S4 and Figure S32B). In literature, the crude extract of *Alternaria alternata* (an endophytic fungus isolated from *Coffea Arabica* L) was reported as moderately cytotoxic towards HELA cells [44].

Besides, the *in vitro* cytotoxicity of alternariol-9-methyl ether (**2**; 9‑methoxy-alternariol), altertoxin II (**4**, also known as stemphyltoxin II), and tenuazonic acid (**5**; also known as l-tenuazonic acid; AAC-toxin) were investigated against A549 and PC3 cells, displaying potent activity of **2** and **4** and moderate activity of **5** (Fig. 5A). Accordingly, the EC_50_ of compounds **2** and **4** were studied showing EC_50_ of 0.73 µg/ml (2.69 µM) and 0.40 µg/ml (1.15 µM) against A549, respectively, meanwhile they showed EC_50_ of 0.17 µg/ml (0.64 µM) and 0.12 µg/ml (0.33 µM) against PC3 (Fig. 5B).

Alternariol and its methyl ether were demonstrated to possess moderate cytotoxic activity on HeLa cell lines [45]. Alternariol 5-O-Me ether isolated from *Setosphaeria* sp., exhibited moderate cytotoxicity against MCF-7, MGC-803, H1975, Huh-7, A549, and HeLa with IC_50_ values ranging from 23.04 to 96.91 µg/mL [46]. In contrast, alternariol was reported to be inactive at 50.0 µM against HepG2 human tumor cell line. Alternatively, altertoxin II showed the highest cytotoxicity to HeLa cells (IC_50_ 0.5 µg/mL) [47], while it showed moderate cytotoxic activity against PC-3 cell lines (IC_50_ value of 14.28 µM) [48], and significant inhibitory activities against the four tumor cell lines MCF-7, HepG-2, NCI-H460 and SF-268 (IC_50_ 1.91–9.67 µg/mL) [48]. Tenuazonic acid derivatives were confirmed to be significant chemotherapeutics against HeLa cells, which in turn supports the finding in our current study [49].

### 3.4 Cytotoxcity mode of action

Mechanistically, the cytotoxicity of alternariol (AOH) (**1**) and its methyl ether derivative (**2**) basically attributed to their capability of breaking DNA by induction of various types of DNA topoisomerase in several *in vitro* cell cultures, have been previously studied [50,51,52]. AOH has been characterized specifically as inhibitor of DNA topoisomerase I and II enzymes, with certain selectivity for the IIa isoform [50,51]. DNA topoisomerases are mainly required during the final stages of DNA replication to facilitate chromosome untangling, DNA condensation and segregation during mitosis, and during translation and general maintenance of the genome [53]. Alternatively, altertoxins I and II (ATX II) (**3, 4**) were found to have even more genotoxic and stronger mutagen activity than alternariol derivatives, which is possibly carried out by inducing DNA adducts as well [54,55]. The cytotoxicity of tenuazonic acid is mainly attributed to its potentiality as protein synthesis inhibitor.

## Declaration of Competing Interest

**Notes:** The authors declare the following competing financial interest: J.S.T. is a co-founder of Centrose (Madison, WI, USA). The authors report no competing interests.

## Appendix Supplementary materials

Image, application 1
