# Supplementary material for: Diverse polyketides from the marine endophytic Alternaria sp. LV52: Structure determination and cytotoxic activities
Source: Biotechnol Rep (Amst). 2021 May 24;33:e00628. doi: 10.1016/j.btre.2021.e00628 (PMC8752877; doi:10.1016/j.btre.2021.e00628)
Supplement: Supplementary file 1 [file mmc1.docx]

**Supporting Information**

**Diverse Polyketides from the Marine Endophytic *Alternaria* sp*.* LV52: Structure Determination and Cytotoxic Activities**

Manar M. Mahmoud,^1,2^ Ahmed S. Abdel-Razek,^2,3^ Hesham Soliman,^1^ Larissa V. Ponomareva,^4,5^ Jon S. Thorson,^4,5^ Khaled A. Shaaban^4,5^ and Mohamed Shaaban^2,6,^*

^1^Pharmacognosy Department, Faculty of Pharmacy, Helwan University, Helwan City-Cairo 11884, Egypt

^2^Organic and Bioorganic Chemistry, Faculty of Chemistry, Bielefeld University, D-33501 Bielefeld, Germany

^3^Microbial Chemistry Department, Division of Genetic Engineering and Biotechnology Research, National Research Centre, El-Buhouth St. 33, Dokki-Cairo 12622, Egypt

^4^Center for Pharmaceutical Research and Innovation, and ^5^Department of Pharmaceutical Sciences, College of Pharmacy, University of Kentucky, Lexington, Kentucky 40536, United States

^6^Chemistry of Natural Compounds Department, Division of Pharmaceutical Industries, National Research Centre, El-Buhouth St. 33, Dokki-Cairo 12622, Egypt

***Correspondence:** mshaaba@gmail.com; Tel: +202-2701728-1550

| **Table of Contents:** | **Page** |
| --- | --- |
| Physico-chemical properties of compounds **1-5** | S3 |
| **Table S1**: ^13^C (125 MHz) and ^1^H (500 MHz) NMR spectroscopic data of alternariol (**1**) and alternariol-9-methyl ether (**2**). | S4 |
| **Table S2**: ^13^C (125 MHz) and ^1^H (500 MHz) NMR spectroscopic data of altertoxin I (**3**) and altertoxin II (**4**) | S4 |
| **Table S3**: ^13^C (125 MHz) and ^1^H (500 MHz) NMR spectroscopic data of tenuazonic acid (**5**) in CDCl_3_ | S5 |
| **Figure S1**: H,H COSY (▬, ↔) and selected HMBC (→) correlations in compounds **1-5**. | S5 |
| **Figure S2-S31:** MS and NMR spectra of compounds **1**-**5** | S6-S35 |
| Figure S32: A) Cytotoxic activity of compounds 1-5 and crude extract against HepG2 cell line. B) Cytotoxic activity of compounds 2-5 and crude extract against HELA cell line. | S36 |
| Table S4: *In vitro* cytotoxicity of compounds 1-5 against HepG2 and HELA cell lines. | S37 |

**Physico-chemical properties of compounds 1-5**

**Alternariol (1)**: C_14_H_10_O_5_ (258), colorless crystals exhibiting pink staining on spraying with anisaldehyde/sulphuric acid. *R*_f_ = 0.33 (CHCl3/5%MeOH). ^1^H NMR (Acetone-*d*_6_, 500 MHz) and ^13^C NMR (Acetone-*d*_6_, 125 MHz), see Table S1. (-)-ESI-MS: *m/z* 256.8 [M-H]^-^.

**Alternariol-9-methyl ether (2)**: C_15_H_12_O_5_ (272), colourless crystals, UV absorbing (254 nm) and blue florescence (366 nm). It exhibited pink staining on spraying with anisaldehyde/sulphuric acid and heating. *R*_f_ = 0.5 (CHCl_3_/5% MeOH). ^1^H NMR (CDCl_3_/CD_3_OD, 500 MHz) and ^13^C NMR (CDCl_3_/CD_3_OD, 125 MHz), see Table S1. (-)-ESI-MS: *m/z* 271 [M-H]^-^.

**Altertoxine I (3)**: C_20_H_16_O_6_ (352) orange crystals, UV absorbing (254 nm), stained brown with anisaldehyde/sulphuric acid spraying and heating. *R*_f_ = 0.38 (CHCl_3_/5% MeOH). ^1^H NMR (CDCl_3_, 500 MHz) and ^13^C NMR (CDCl_3_, 125 MHz), see Table S2. (-)-ESI-MS: *m/z* 351 [M-H]^-^.

**Altertoxine II (4)**: C_20_H_14_O_6_ (350), brownish orange solid, showing orange staining by treatment with anisaldehyde/sulphuric acid and heating. It is strong UV absorbing (254 nm) and green fluorescence (365 nm). *R*_f_ = 0.29 (CHCl_3_/5 %MeOH). ^1^H NMR (Acetone-*d*_6_, 500 MHz) and ^13^C NMR (Acetone-*d*_6_, 125 MHz), see Table S2. (+)-ESI-MS *m/z* 351 [M+H]^+^.

**Tenuazonic acid (5)**: C_10_H_15_NO_3_ (197), colourless solid. Strong UV absorbing (254 nm) as well as strong blue florescence (365 nm). *R*_f_ = 0.35 (CHCl_3_/5 %MeOH). ^1^H NMR (CDCl_3_, 500 MHz) and ^13^C NMR (CDCl_3_, 125 MHz), see Table S3. (-)-ESI-MS: *m/z* 195.8 [M-H]^-^.

Table S1: ^13^C (125 MHz) and ^1^H (500 MHz) NMR spectroscopic data of alternariol (1) and alternariol-9-methyl ether (2).

| **Position** | **Alternariol (1)^a^** | | **Alternariol-9-methyl ether (2)^b^** | |
| --- | --- | --- | --- | --- |
|  | **δ_C_** | **δ_H_** | **δ_C_** | **δ_H_** |
| 1 | 138.5 |  | 138.4 |  |
| 1-CH_3_ | 24.9 | 2.64 | 25.3 | 2.70 (s) |
| 2 | 117.4 | 6.66 (d, 2.7) | 117.6 | 6.62 (d, 2.7) |
| 3 | 158.3 |  | 158.2 |  |
| 4 | 101.8 | 6.57 (d, 2.6) | 102.0 | 6.60 (d, 2.6 Hz) |
| 4a | 152.9 |  | 153.0 |  |
| 6 | 165.0 |  | 165.7 |  |
| 7a | 98.3 |  | 98.9 |  |
| 7 | 164.5 |  | 164.5 |  |
| 8 | 101.0 | 6.32 (d, 2.1) | 98.6 | 6.45 (d, 2.2 Hz) |
| 9 | 165.1 |  | 166.6 |  |
| 9-OCH_3_ |  |  | 55.4 | 3.84 (s) |
| 10 | 104.3 | 7.23 (d, 2.1) | 104.0 | 7.19 (d, 2.3 Hz) |
| 10a | 138.2 |  | 138.3 |  |
| 10b | 109.7 |  | 109.8 |  |

^a)^Acetone-*d*_6_ ; ^b)^ CDCl_3_/CD_3_OD

Table S2: ^13^C (125 MHz) and ^1^H (500 MHz) NMR spectroscopic data of altertoxin I (3) and altertoxin II (4).

| Position | Altertoxin I (**3**) (CDCl_3_) | | Altertoxin II (**4**) (Acetone-*d*_6_) | |
| --- | --- | --- | --- | --- |
|  | δ_C_ | δ_H_ | δ_C_ | δ_H_ |
| 1 | 205.0 |  | 205.0 |  |
| 2 | 34.0 | 3.11 (ddd, 17.3, 14.4, 4.8),  2.64 (ddd, 17.2, 3.9, 2.7) | 33.0 | 3.12(ddd, 17.3, 14.1, 5.0),  2.65 (ddd, 17.2, 3.9, 2.7) |
| 3 | 34.5 | 2.95 (ddd, 14.4, 4.8, 2.7),  2.36 (td, 14.4, 3.9) | 32.3 | 2.84 (ddd, 13.4, 5.0, 2.6),  2.47 (td, 14.4, 3.9) |
| 4 | 69.2 |  | 67.9 |  |
| 4a | 139.1 |  | 140.3 |  |
| 5 | 122.5 |  | 123.1 |  |
| 6 | 132.5 | 7.76 (d) | 132.7 | 8.00 (d, 8.8) |
| 7 | 119.5 | 7.02 (d, 8.8) | 118.4 | 6.93 (d, 8.8) |
| 8 | 162.3 |  | 162.2 |  |
| 8-OH |  | 12.64 |  | 12.66 |
| 8a | 113.8 |  | 113.8 |  |
| 1' | 202.1 |  | 198.0 |  |
| 2' | 47.8 | 3.05 (m), 2.86 (dd, 16.0, 11.9) | 52.7 | 3.60 (dd, 3.7, 0.8) |
| 3' | 66.1 | 4.70 (m) | 56.1 | 4.31 (d, 3.7) |
| 4' | 51.9 | 3.02 (m) | 44.8 | 3.50 (s) |
| 4a' | 135.5 |  | 135.6 |  |
| 5' | 124.1 |  | 124.8 |  |
| 6' | 132.7 | 7.77 (d, 8.8) | 132.2 | 7.91 (d, 8.8) |
| 7' | 117.6 | 6.96 (dd, 8.7, 0.9) | 116.6 | 6.93 (dd, 8.8) |
| 8' | 162.0 |  | 162.7 |  |
| 8'-OH |  | 12.27 |  | 11.95 |
| 8a' | 116.9 |  | 114.2 |  |

Table S3. ^13^C (125 MHz) and ^1^H (500 MHz) NMR spectroscopic data of tenuazonic acid (5) in CDCl_3_.

| Position | δ_C_ | δ_H_ |
| --- | --- | --- |
| 1 |  | 13.07 (brs) |
| 2 | 175.7 |  |
| 3 | 102.5 |  |
| 4 | 195.8 |  |
| 4-OH |  | 7.99 (s) |
| 5 | 67.3 | 3.75 (d, 3.4) |
| 6 | 36.9 | 1.91 (m) |
| 7 | 23.5 | 1.17 (tdd, 14.5, 12.1, 7.1),  1.31 (dqd, 14.7, 7.4, 4.0) |
| 8 | 11.6 | 0.83 (t, 7.4) |
| 9 | 15.7 | 0.97 (d, 7.0) |
| 10 | 184.2 |  |
| 11 | 19.4 | 2.39 (s) |

Figure S1. H,H COSY (▬, ↔) and selected HMBC (→) correlations in compounds 1-5.


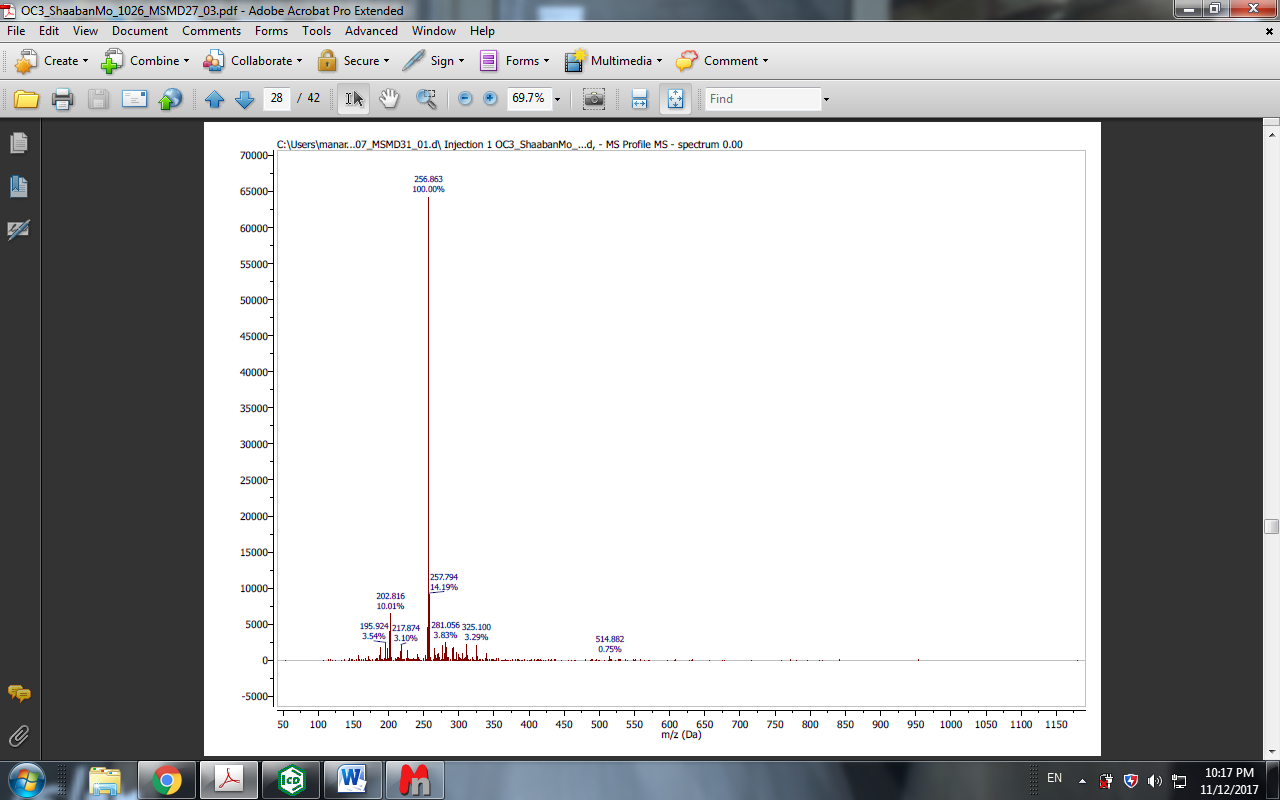


**[M-H]^-^**

**Figure S2:** (-)-ESIMS spectrum of Alternariol (**1)**

**Figure S3:** ^1^H NMR (Acetone-*d*_6_, 500 MHz) spectrum of Alternariol (**1**)

**Figure S4:**^13^C NMR (Acetone-*d*_6_, 125 MHz) spectrum of Alternariol (**1**)

**Figure S5:** H,H-COSY spectrum (Acetone-*d*_6_, 500 MHz) of Alternariol (**1**)

**Figure S6:** HMQC spectrum (Acetone-*d*_6_, 500 MHz) of Alternariol (**1**)

**Figure S7:** HMBC spectrum (Acetone-*d*_6_, 500 MHz) of Alternariol (**1**)


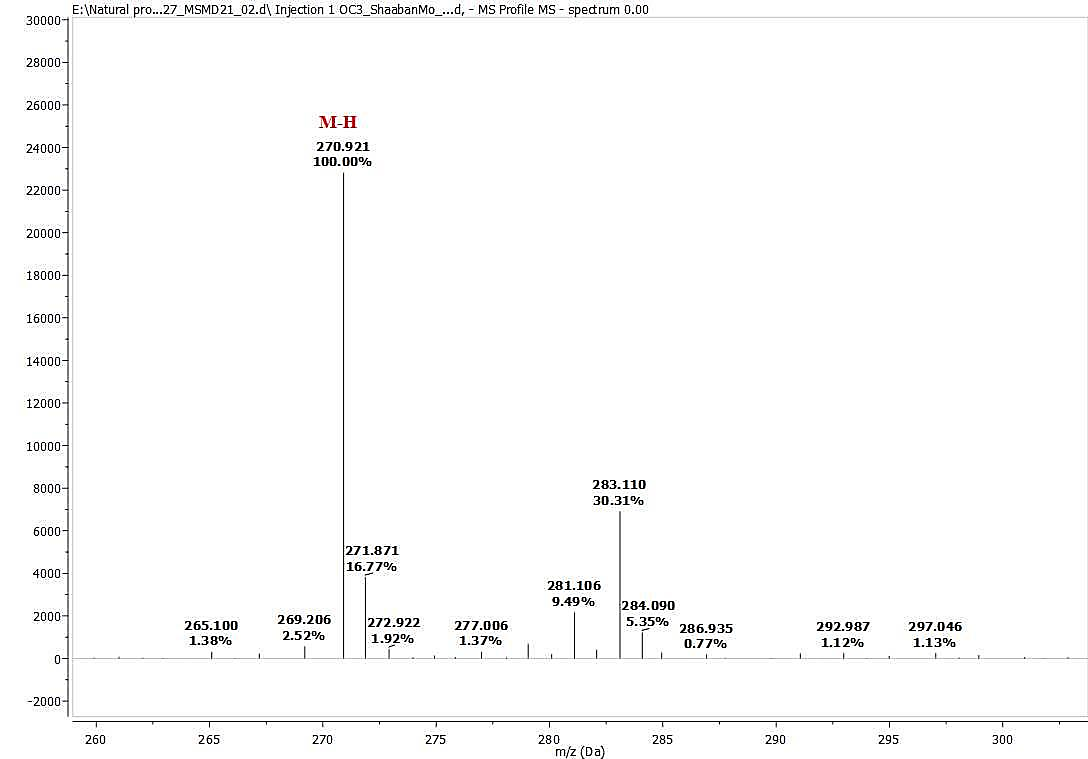


Figure S8. (-)-ESIMS spectrum of 9-Methoxy-alternariol (2)

**Figure S9**: ^1^H NMR (CD_3_OD/CDCl_3_, 500 MHz) spectrum of 9-methoxy-alternariol (**2**)

**Figure S10:** ^13^C NMR (CD_3_OD/CDCl_3_, 125 MHz) spectrum of 9-Methoxy-alternariol (**2**)

**Figure S11:** H,H-COSY spectrum (CD_3_OD/CDCl_3_, 500 MHz) of 9-Methoxy-alternariol (**2**)

**Figure S12.** HMQC spectrum (CD_3_OD/CDCl_3_, 500 MHz) of 9-Methoxy-alternariol (**2**)

**Figure S13.** HMBC spectrum (CD_3_OD/CDCl_3_, 500 MHz) of 9-Methoxy-alternariol (**2**)


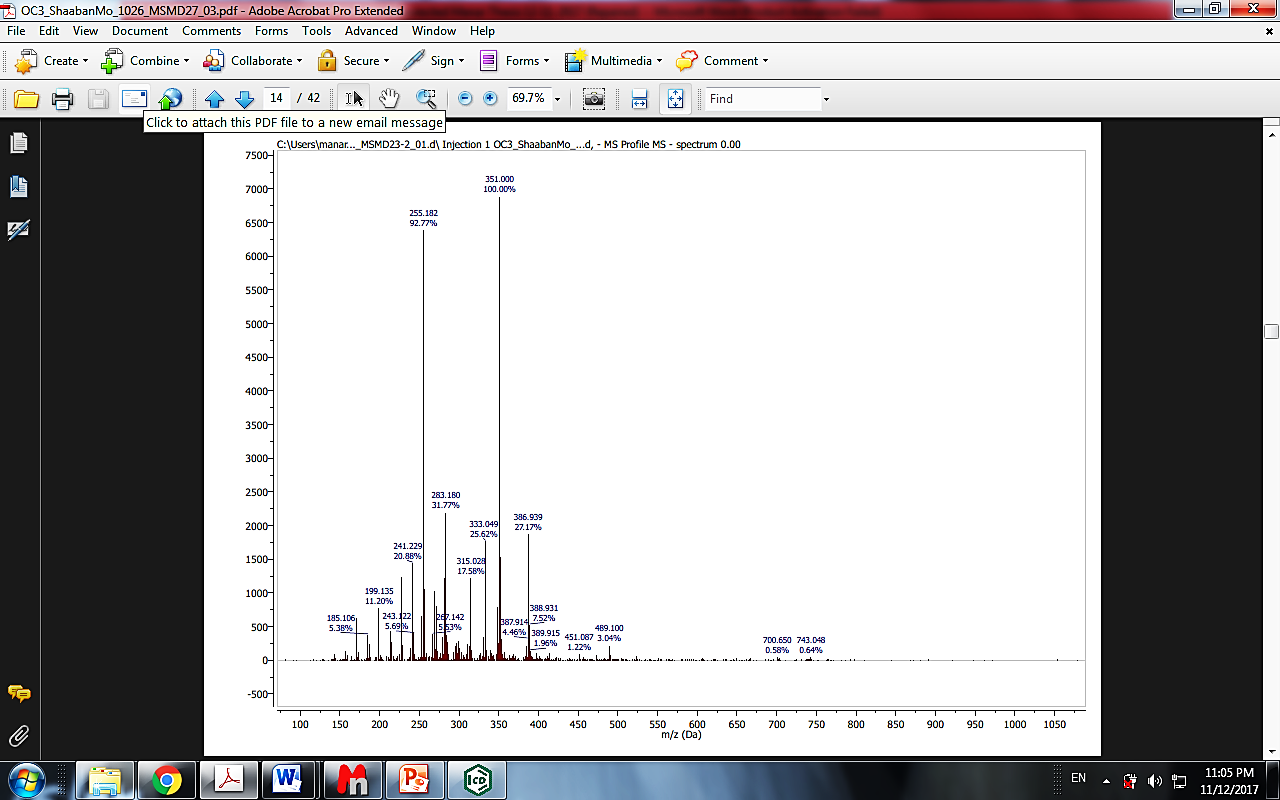


**351 ([M-H]^-^, 100)**

**Figure S14: (-)-**ESIMS spectrum of Altertoxin I **(3)**

CH_2_-12

CH_2_-11

CH-12b

CH_2_-2

**Figure S15:** ^1^H NMR (CDCl_3_, 500 MHz) spectrum of Altertoxin I (**3**)

**Figure S16.** ^13^C NMR (CDCl_3_, 125 MHz) spectrum of Altertoxin I (**3**)

**Figure S17:** DEPT spectrum (CDCl_3_, 125 MHz) of Altertoxin I (**3**)

**Figure S18:** H,H-COSY spectrum (CDCl_3_, 500 MHz) of Altertoxin I (**3**)

**Figure S19:** HMQC spectrum (CDCl_3_, 500 MHz) of Altertoxin I (**3**)

**Figure S20:** HMBC spectrum (CDCl_3_, 500 MHz) of Altertoxin I (**3**)

CH_2_-12

CH_2_-11

CH_2_-11

CH_2_-12

**Figure S21:** ^1^H NMR (Acetone-*d*_6_, 500 MHz) spectrum of Altertoxin II (**4**)

**Figure S22:** ^13^C NMR spectrum (Acetone-*d*_6_, 125 MHz) of Altertoxin II (**4**)

**Figure S23:** H-H COSY spectrum (Acetone-*d*_6_, 500 MHz) of Alteroxine II (**4)**

**Figure S24:** HMQC spectrum (Acetone-*d*_6_, 500 MHz) of Alteroxine II (**4)**

**Figure S25:** HMBC spectrum (Acetone-*d*_6_, 500 MHz) of Alteroxine II (**4**)

**[M-H]^-^**

**Figure S26:** (-)-ESIMS spectrum of Tenuazonic acid (**5**)

**Figure S27:** ^1^H NMR (CDCl_3_, 500 MHz) spectrum of Tenuazonic acid (**5**)

**Figure S28:** ^13^C NMR (CDCl_3_, 125 MHz) spectrum of Tenuazonic acid (**5**)

**Figure S29:** H,H-COSY spectrum (CDCl_3_, 500 MHz) of Tenuazonic acid (**5**)

**Figure S30:** HMQC spectrum (CDCl_3_, 500 MHz) of Tenuazonic acid (**5**)

**Figure S31:** HMBC spectrum (CDCl_3_, 500 MHz) of Tenuazonic acid (**5**)


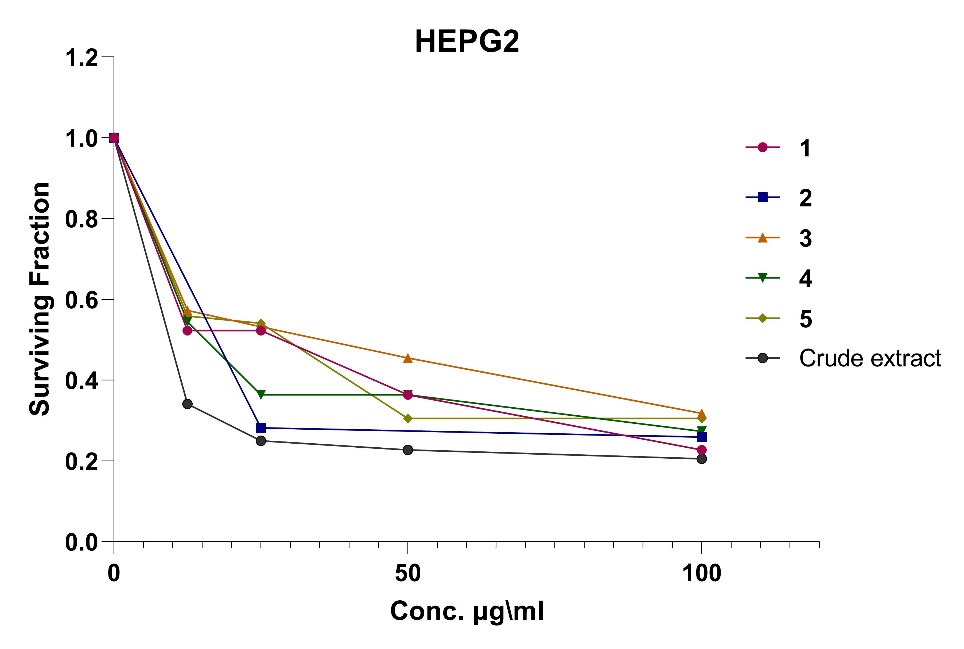

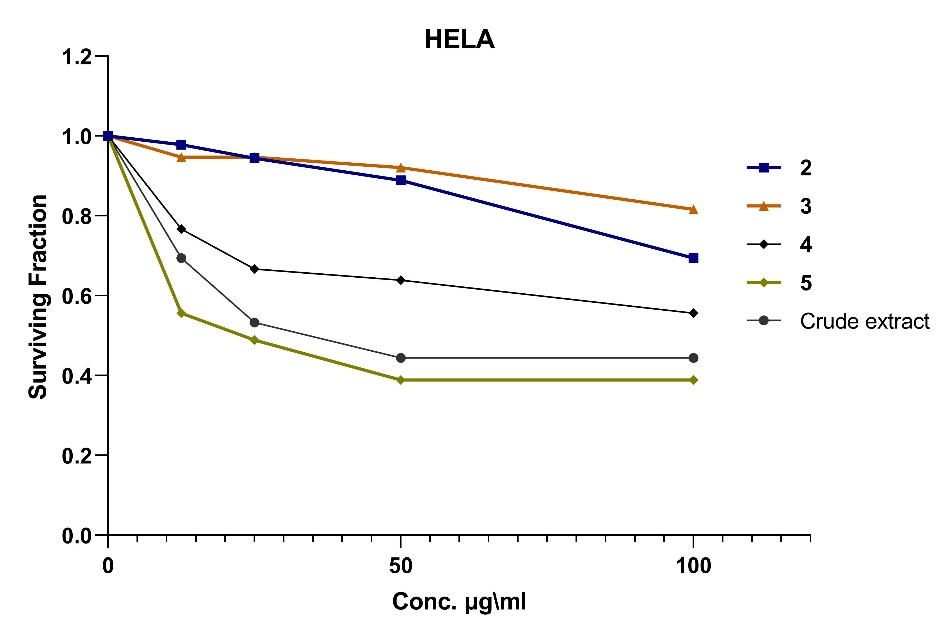


**A)**

**B)**

**Figure S32: A)** Cytotoxic activity of compounds **1-5** and crude extract against HepG2 cell line. **B)** Cytotoxic activity of compounds 2-5 and crude extract against HELA cell line. For the summary of EC_50_, see Table S1 and Figure 5. **HEPG2:** EC_50_ for compounds [**1 (**28 μg/ml), **2** **(**9.8 μg/ml), **3 (**34 μg/ml), **4 (**15 μg/ml), **5 (**28 μg/ml), **crude extract** (9.34 μg/ml) and **Doxorubicin** (4.28 μg/ml)]. **HELA:** EC_50_ for compounds **[2-4** (>50 μg/ml)**, 5 (**21.5 μg/ml), **Crude extract** (34 μg/ml) and **Doxorubicin** (1.45 μg/ml)]. Compound **1** was not tested against HELA cell line due to the lack of material.

Table S4: *In vitro* cytotoxicity of compounds 1-5 against HepG2 and HELA cell lines.

| **Compound\ Ext** | ***HepG2*** | | | ***HELA*** | | | | | |
| --- | --- | --- | --- | --- | --- | --- | --- | --- | --- |
|  | SF | **μg\ml** | **EC_50_ (μg\ml)** | **SF** | **μg\ml** | | | **EC_50_ (μg\ml)** | |
| **1** | 1.000 | 0.000 | 28.00 | - | | | | | |
|  | 0.523 | 12.500 |  |  |  |  |  |  |  |
|  | 0.523 | 25.000 |  |  |  |  |  |  |  |
|  | 0.364 | 50.000 |  |  |  |  |  |  |  |
|  | 0.227 | 100.000 |  |  |  |  |  |  |  |
| **2** | 1.000 | 0.000 | 9.80 | 1.000 | | | 0.000 | | 88.20 |
|  | 0.359 | 12.500 |  | 0.978 | | | 12.500 | |  |
|  | 0.282 | 25.000 |  | 0.944 | | | 25.000 | |  |
|  | 0.282 | 50.000 |  | 0.889 | | | 50.000 | |  |
|  | 0.259 | 100.000 |  | 0.694 | | | 100.000 | |  |
| **3** | 1.000 | 0.000 | 34.00 | 1.000 | | | 0.000 | | 94.60 |
|  | 0.573 | 12.500 |  | 0.947 | | | 12.500 | |  |
|  | 0.532 | 25.000 |  | 0.947 | | | 25.000 | |  |
|  | 0.455 | 50.000 |  | 0.921 | | | 50.000 | |  |
|  | 0.318 | 100.000 |  | 0.816 | | | 100.000 | |  |
| **4** | 1.000 | 0.000 | 15.00 | 1.000 | | | 0.000 | | 62.00 |
|  | 0.545 | 12.500 |  | 0.767 | | | 12.500 | |  |
|  | 0.364 | 25.000 |  | 0.667 | | | 25.000 | |  |
|  | 0.364 | 50.000 |  | 0.639 | | | 50.000 | |  |
|  | 0.273 | 100.000 |  | 0.556 | | | 100.000 | |  |
| **5** | 1.000 | 0.000 | 28.80 | 1.000 | | | 0.000 | | 21.50 |
|  | 0.559 | 12.500 |  | 0.556 | | | 12.500 | |  |
|  | 0.541 | 25.000 |  | 0.489 | | | 25.000 | |  |
|  | 0.305 | 50.000 |  | 0.389 | | | 50.000 | |  |
|  | 0.305 | 100.000 |  | 0.389 | | | 100.000 | |  |
| **Crude extract** | 1.000 | 0.000 | 9.34 | 1.000 | | | 0.000 | | 34.00 |
|  | 0.341 | 12.500 |  | 0.694 | | | 12.500 | |  |
|  | 0.250 | 25.000 |  | 0.533 | | | 25.000 | |  |
|  | 0.227 | 50.000 |  | 0.444 | | | 50.000 | |  |
|  | 0.205 | 100.000 |  | 0.444 | | | 100.000 | |  |
| **Doxorubicin** | - | - | 4.28 | - | | - | | | 1.45 |
